# Central Pontine Myelinolysis: A Rare and Life-Threatening Adverse Effect of Clobazam and Quetiapine

**DOI:** 10.7759/cureus.60007

**Published:** 2024-05-09

**Authors:** Gautam N Bedi, Sourya Acharya, Sunil Kumar, Smruti A Mapari, Rucha Sawant

**Affiliations:** 1 Medicine, Jawaharlal Nehru Medical College, Datta Meghe Institute of Higher Education and Research, Wardha, IND; 2 Obstetrics and Gynecology, Jawaharlal Nehru Medical College, Datta Meghe Institute of Higher Education and Research, Wardha, IND

**Keywords:** mri imaging, neurological disorder, quetiapine, clobazam, central pontine myelinolysis

## Abstract

Central pontine myelinolysis (CPM) is a rare neurological disorder characterized by demyelination within the central portion of the pons. While hyponatremia is a well-known precipitating factor, other etiologies, including medication use, have been reported. We present a case of a 69-year-old male with a history of obsessive-compulsive disorder, stroke, and type 2 diabetes mellitus who developed confusion, altered sensorium, and weakness in all four limbs. An MRI brain imaging revealed characteristic findings suggestive of CPM. Despite normal serum sodium levels, discontinuation of clobazam and quetiapine, medications taken by the patient, led to clinical improvement. This case underscores the importance of considering medication-induced CPM in the differential diagnosis of patients presenting with neurological symptoms, even in the absence of electrolyte abnormalities.

## Introduction

Central pontine myelinolysis (CPM) is a rare neurological disorder characterized by demyelination within the central portion of the pons, often resulting in severe neurological deficits. Originally described by Adams et al. in 1959, CPM was primarily associated with rapid correction of hyponatremia, particularly in the setting of chronic alcoholism or liver transplantation [1]. However, it has become increasingly recognized that CPM can also occur in the absence of significant electrolyte disturbances, particularly in patients with predisposing factors such as alcoholism, liver disease, malnutrition, and certain medications [2]. One such medication implicated in the development of CPM is clobazam, a benzodiazepine derivative commonly used in the management of various neurological and psychiatric disorders, including epilepsy and anxiety disorders. While clobazam is generally well-tolerated, there have been reported cases of CPM associated with its use, particularly in patients with underlying risk factors such as electrolyte imbalances or liver dysfunction [3].

Similarly, quetiapine, an atypical antipsychotic agent widely prescribed for the treatment of schizophrenia, bipolar disorder, and major depressive disorder, has also been linked to the development of CPM. Although the exact mechanisms underlying quetiapine-induced CPM remain unclear, it is hypothesized that its anticholinergic effects and potential for causing electrolyte disturbances may contribute to myelinolysis in susceptible individuals [4]. Given the increasing recognition of medication-induced CPM, clinicians must remain vigilant for neurological symptoms in patients receiving these medications, particularly those with underlying risk factors. Prompt recognition and cessation of the offending agents and supportive management are essential in preventing further neurological deterioration and optimizing patient outcomes.

## Case presentation

A 69-year-old male presented to the emergency department with chief complaints of confusion, altered sensorium, and weakness in all four limbs, persisting for six hours. His medical history revealed a diagnosis of obsessive-compulsive disorder over the past three months, managed with a stable regimen of clobazam 10 mg twice daily and quetiapine 50 mg once daily. Additionally, he had a known history of stroke and type 2 diabetes mellitus, for which he was prescribed voglibose GM3 twice daily. Random blood sugar levels obtained in the emergency department were 110 mg/dL, falling within the normal range. There was no recent history of illness, head trauma, or significant electrolyte abnormalities reported.

Upon physical examination, the patient appeared drowsy. Vital signs revealed a heart rate of 88 beats per minute, blood pressure of 120/80 mmHg, and oxygen saturation of 99% on room air. Neurological examination showed normal tone with 4/5 power in both lower limbs and bilateral plantar flexor response.

The patient's medications, clobazam and quetiapine, were considered potential contributors to the development of CPM. Consequently, both medications were promptly discontinued. Blood investigations were ordered, and the results are outlined in Table 1.

**Table 1:** Biochemical parameters during the course of hospitalization

Investigations	Reference range	Value on admission	Value on discharge
Serum sodium (mmol/L)	135-147	142	140
Serum potassium (mmol/L)	3.5-5.1	4.29	4.5
Serum calcium (mg/dL)	4.5-5.6	4.24	4.3
Urine sodium (mmol/L)	75-200	154	160
Urine potassium (mmol/L)	10-20	16	18
Urine calcium (mg/dL)	4.6-5.3	4.8	5.2

**Figure 1:**
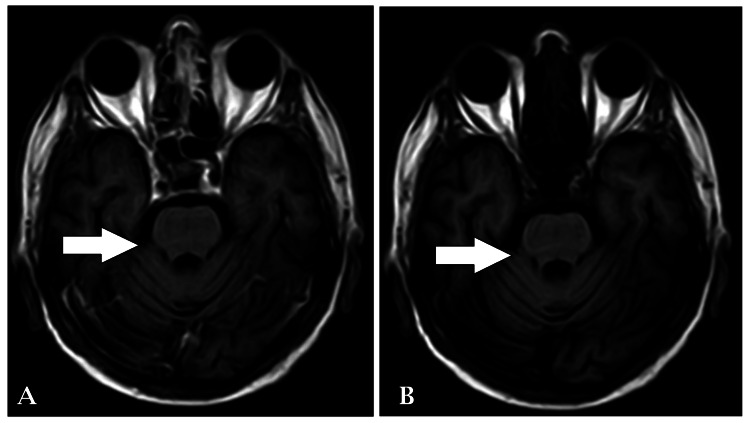
T1WI and T1+C axial section of brain showing non-enhancing lesion in pons T1WI: T1-weighted imaging; T1+C: T1-weighted imaging with contrast

**Figure 2:**
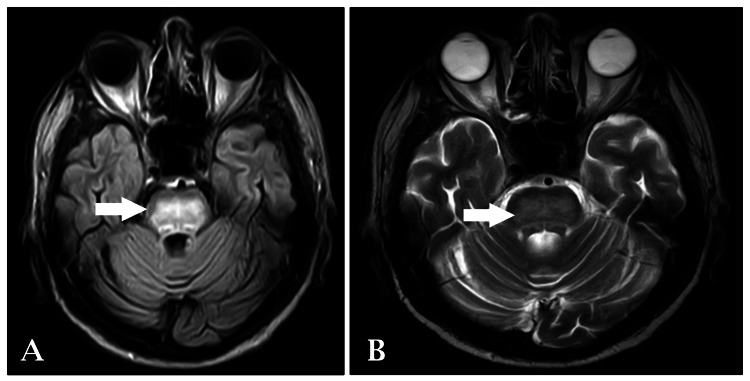
T2-weighted and FLAIR axial section of brain showing hyperintense lesion in pons extending to both sides of the midline FLAIR: fluid-attenuated inversion-recovery

**Figure 3:**
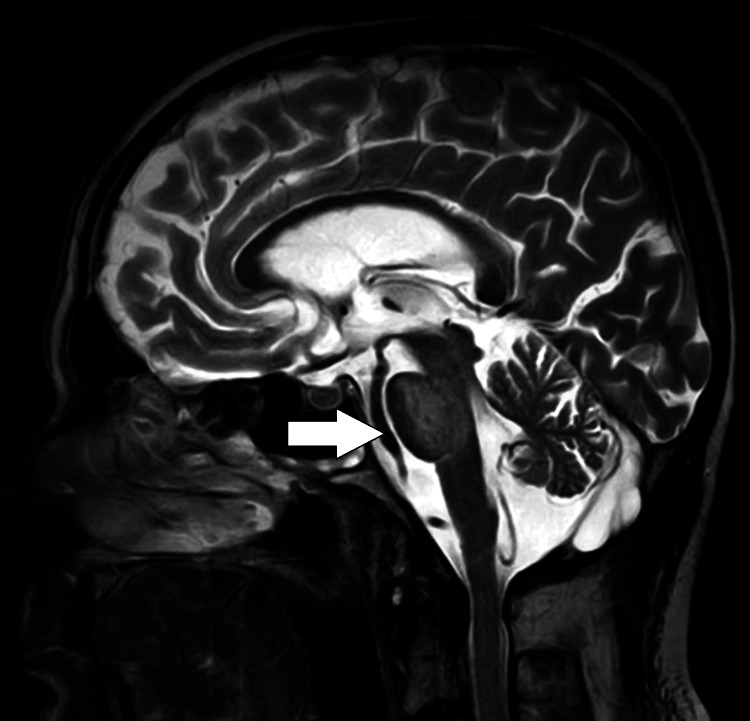
T2WI sagittal section of brain showing hyperintense lesion in pons T2WI: T2-weighted imaging

An MRI brain imaging contrast with spectroscopy revealed a non-enhancing altered signal intensity noted in the pons, extending to both sides of the midline. The lesion appeared isointense in T1-weighted images (Figure 1) and hyperintense in T2-weighted/fluid-attenuated inversion-recovery (FLAIR) images (Figures 2-3), with no restriction on diffusion-weighted imaging (DWI) and high signal on apparent diffusion coefficient (ADC), suggestive of osmotic demyelination. Age-related atrophic changes were noted in various brain structures, along with small vessel ischemic changes observed in bilateral subcortical and periventricular deep white matter.

**Figure 4:**
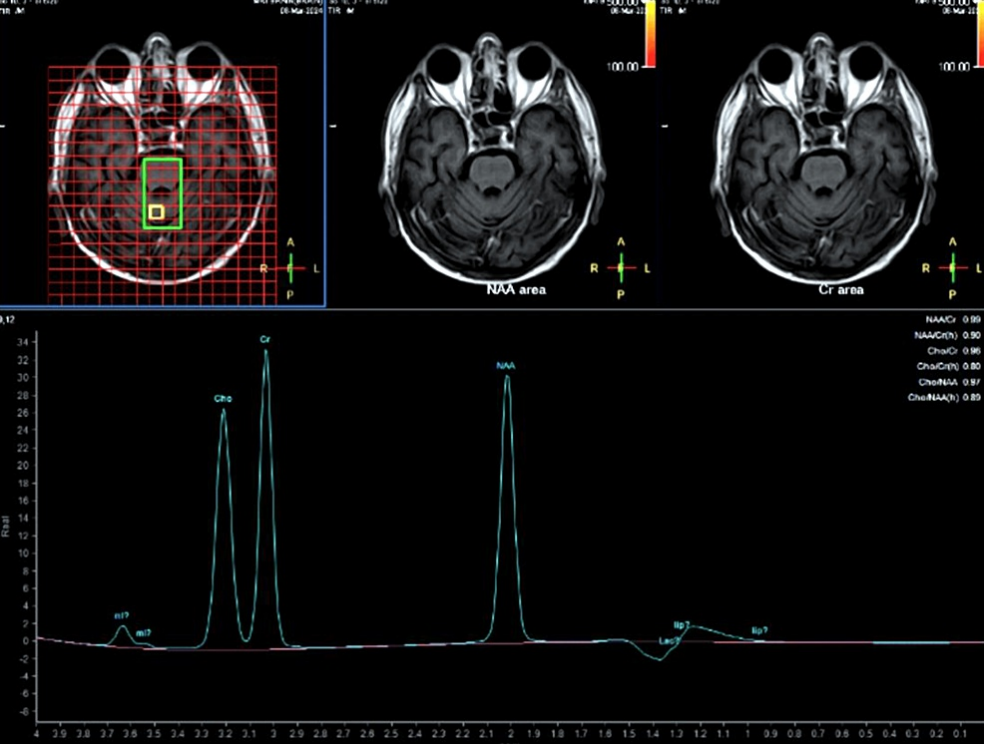
Magnetic resonance spectroscopy showing the raised NAA peak and Cr peak with decreased NAA/Cr ratio (0.99) NAA: N-acetyl aspartate; Cr: creatinine

The spectroscopy showed elevated N-acetyl aspartate (NAA) and creatinine (Cr) peak levels with a decreased NAA/Cr ratio (0.99) (Figure 4).

Serum sodium levels, as well as urinary sodium and potassium levels, were within normal limits. This effectively ruled out the possibility of the syndrome of inappropriate secretion of antidiuretic hormone (SIADH). Despite diabetes mellitus being a triggering factor for CPM, the patient's diabetes was well-controlled, leading to the conclusion of a direct association between clobazam and quetiapine and the development of CPM.

The treatment focused on providing symptomatic relief and supportive management, including antibiotics, analgesics, antacids, antiemetics, antidiabetic medications, and hydration and electrolyte monitoring. Alternative antipsychotic agents with a lower propensity for metabolic side effects were considered. The patient's care involved multiple specialties utilizing a multidisciplinary approach, leading to a positive response to treatment.

## Discussion

CPM is a rare and potentially life-threatening neurological disorder characterized by demyelination within the central portion of pons. It typically presents with a spectrum of neurological symptoms, including confusion, altered sensorium, and weakness, as observed in our patient [5]. While the exact pathogenesis of CPM remains incompletely understood, it is commonly associated with rapid correction of hyponatremia, particularly in patients with chronic alcoholism, liver disease, or malnutrition [2]. In our case, the patient developed CPM despite normal serum sodium levels and the absence of electrolyte abnormalities. This is consistent with reports in the literature suggesting that other factors, such as medication use, may contribute to the development of CPM [6]. Indeed, both clobazam and quetiapine, medications taken by our patient, have been implicated in the pathogenesis of CPM [7,8].

Clobazam is a benzodiazepine commonly used in the management of various neurological disorders, including epilepsy and anxiety disorders. While it is generally well-tolerated, there have been reports linking clobazam use with the development of CPM, particularly in elderly patients or those with underlying risk factors [7]. Similarly, quetiapine, an atypical antipsychotic agent, has been associated with CPM in several case reports [8]. The mechanism by which these medications contribute to the development of CPM is not fully elucidated. However, it is speculated that they may disrupt the osmotic balance within the brain, leading to cellular swelling and subsequent demyelination [9]. Additionally, patients with pre-existing neurological conditions, such as stroke, as seen in our patient, may be at increased risk of developing CPM when exposed to these medications [10].

The diagnosis of CPM in our patient was supported by characteristic findings on MRI brain imaging, including hyperintensity on T2-weighted/FLAIR images and the absence of diffusion restriction on DWI. Furthermore, spectroscopy revealed biochemical alterations consistent with demyelination, further supporting the diagnosis [11]. CPM management primarily involves discontinuing the offending agent and supportive care to alleviate symptoms. In our case, the cessation of clobazam and quetiapine was promptly initiated, leading to clinical improvement. Supportive measures were also provided, including hydration, electrolyte monitoring, and symptomatic relief.

## Conclusions

In conclusion, our case highlights the importance of considering medication-induced CPM as a potential complication in patients presenting with neurological symptoms, even in the absence of classical risk factors such as hyponatremia. The prompt recognition of CPM and discontinuation of the offending medications, clobazam and quetiapine in this instance, are paramount to prevent further neurological deterioration. While the pathogenesis of medication-induced CPM remains incompletely understood, it underscores the need for heightened vigilance among clinicians prescribing medications with potential neurotoxic effects, particularly in patients with underlying neurological comorbidities. Additionally, our case underscores the value of a multidisciplinary approach to patient care involving various specialties to ensure comprehensive management and favorable outcomes. Further research is warranted to elucidate the mechanisms underlying medication-induced CPM and to develop strategies for its prevention and management.
